# Primary Cutaneous Epithelioid Hemangioendothelioma of the Scalp With Pulmonary, Hepatic, and Osseous Metastases: A Case Report

**DOI:** 10.7759/cureus.116186

**Published:** 2026-09-13

**Authors:** Nada Tahri, Kaoutar Belharti, Rania Bouabdellaoui, Soufia El Ouardani, Nassiba Zerrouki, Said Afqir, Nada Zizi

**Affiliations:** 1 Department of Dermatology and Venereology, Mohammed VI University Hospital, Oujda, MAR; 2 Department of Medical Oncology, Mohammed VI University Hospital, Oujda, MAR; 3 Laboratory of Epidemiology, Clinical Research, and Public Health, Faculty of Medicine and Pharmacy of Oujda, Mohammed First University, Oujda, MAR

**Keywords:** cutaneous neoplasm, epithelioid hemangioendothelioma, metastasis, scalp, vascular tumor

## Abstract

Epithelioid hemangioendothelioma (EHE) is a rare vascular neoplasm of intermediate malignant potential. Primary cutaneous involvement is exceedingly uncommon, and scalp localization is exceptional. We report the case of a 61-year-old woman with primary cutaneous EHE of the scalp that progressed to synchronous pulmonary, hepatic, and osseous metastases. The diagnosis was established based on clinicopathological, immunohistochemical, and imaging findings. The patient initially received weekly paclitaxel; however, disease progression after six months prompted a switch to doxorubicin, with clinical stability at the latest follow-up. This case highlights the potential for aggressive clinical behavior despite the rarity of this tumor and emphasizes the importance of complete surgical excision, long-term follow-up, and multidisciplinary management.

## Introduction

Epithelioid hemangioendothelioma (EHE) is a rare vascular neoplasm of intermediate malignant potential arising from vascular endothelial cells [1]. It is characterized by a highly variable clinical course, ranging from indolent localized disease to aggressive metastatic dissemination. EHE most commonly involves the liver, lungs, bones, and deep soft tissues, whereas primary cutaneous involvement is exceedingly uncommon, with scalp localization being exceptionally rare [1,2]. Most EHE cases harbor a WWTR1::CAMTA1 gene fusion, while a minority of cases harbor a YAP1::TFE3 fusion.

Compared with EHE arising in deep soft tissues or visceral organs, primary cutaneous EHE generally has a more favorable prognosis because localized lesions can often be successfully managed by complete surgical excision. However, local recurrence and distant metastases may still occur, emphasizing the need for long-term follow-up [1].

Although isolated cases of primary cutaneous EHE of the scalp associated with lymph node metastasis, metastatic scalp involvement from EHE arising at other sites, and primary calvarial EHE presenting as scalp swelling have been reported, to our knowledge, no previous report has described a primary cutaneous scalp EHE associated with pulmonary, hepatic, and osseous metastases [3-5]. We report this exceptionally rare presentation to highlight its aggressive clinical behavior, the diagnostic challenges it poses, and the importance of early recognition and multidisciplinary management.

## Case presentation

A 61-year-old woman with a medical history of hypertension and type 2 diabetes mellitus initially presented to a private dermatologist three years before referral to our department with a 1-cm ulcerated exophytic lesion on the scalp. The lesion was completely excised, and histopathological examination established the diagnosis of EHE with positive surgical margins. The patient did not return for postoperative follow-up, and consequently no additional treatment was performed.

Approximately one year later, she noticed a gradual recurrence of the lesion at the site of the previous excision but did not seek further medical attention. Eighteen months later, corresponding to six months before presentation to our department, she developed a persistent cough and was referred to the Department of Thoracic Surgery for further evaluation. Contrast-enhanced thoracoabdominal computed tomography (CT) revealed multiple pulmonary and hepatic lesions suspicious for metastatic disease, prompting further evaluation with whole-body fluorine-18 fluorodeoxyglucose positron emission tomography/CT (18F-FDG PET/CT) (Figures 1, 2).

**Figure 1:**
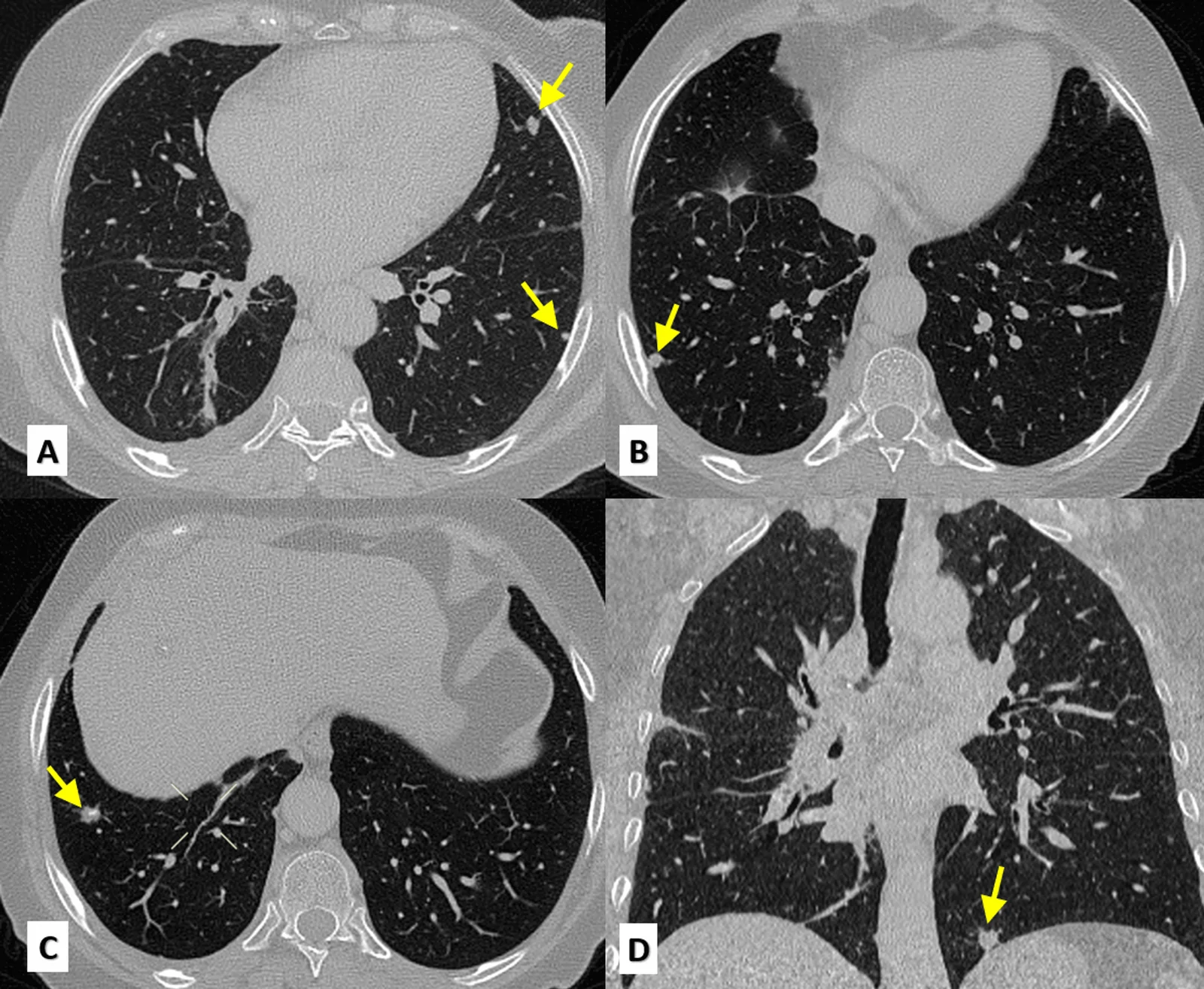
Contrast-enhanced chest CT images in the lung window (A-C) Axial and (D) coronal views showing multiple bilateral pulmonary nodules involving the lower lobes and the left upper lobe (arrows), several demonstrating intralesional calcifications, consistent with pulmonary metastatic disease CT: computed tomography

**Figure 2:**
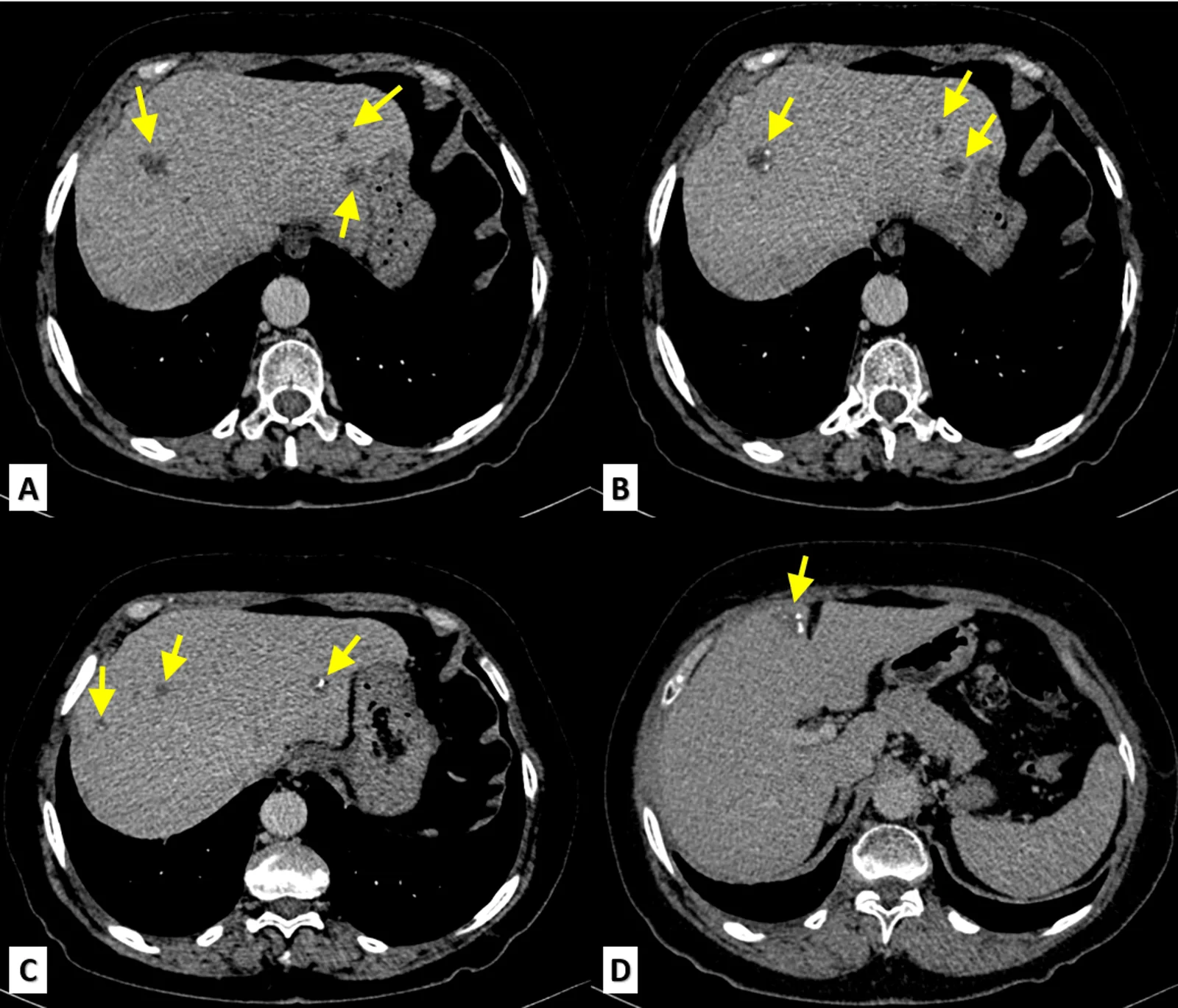
Contrast-enhanced abdominal CT scan (A-D) Axial views demonstrating multiple hypodense hepatic lesions involving segments II, IV, and VIII (arrows). Several lesions show intralesional calcifications, consistent with hepatic metastases CT: computed tomography

The patient was subsequently referred to our dermatology department to investigate a possible cutaneous primary lesion. Dermatological examination revealed a well-demarcated, indurated, ulcerated, infiltrated plaque measuring approximately 6 cm in diameter on the vertex of the scalp. The lesion exhibited erythematous, blackish, and suppurative areas (Figure 3).

**Figure 3:**
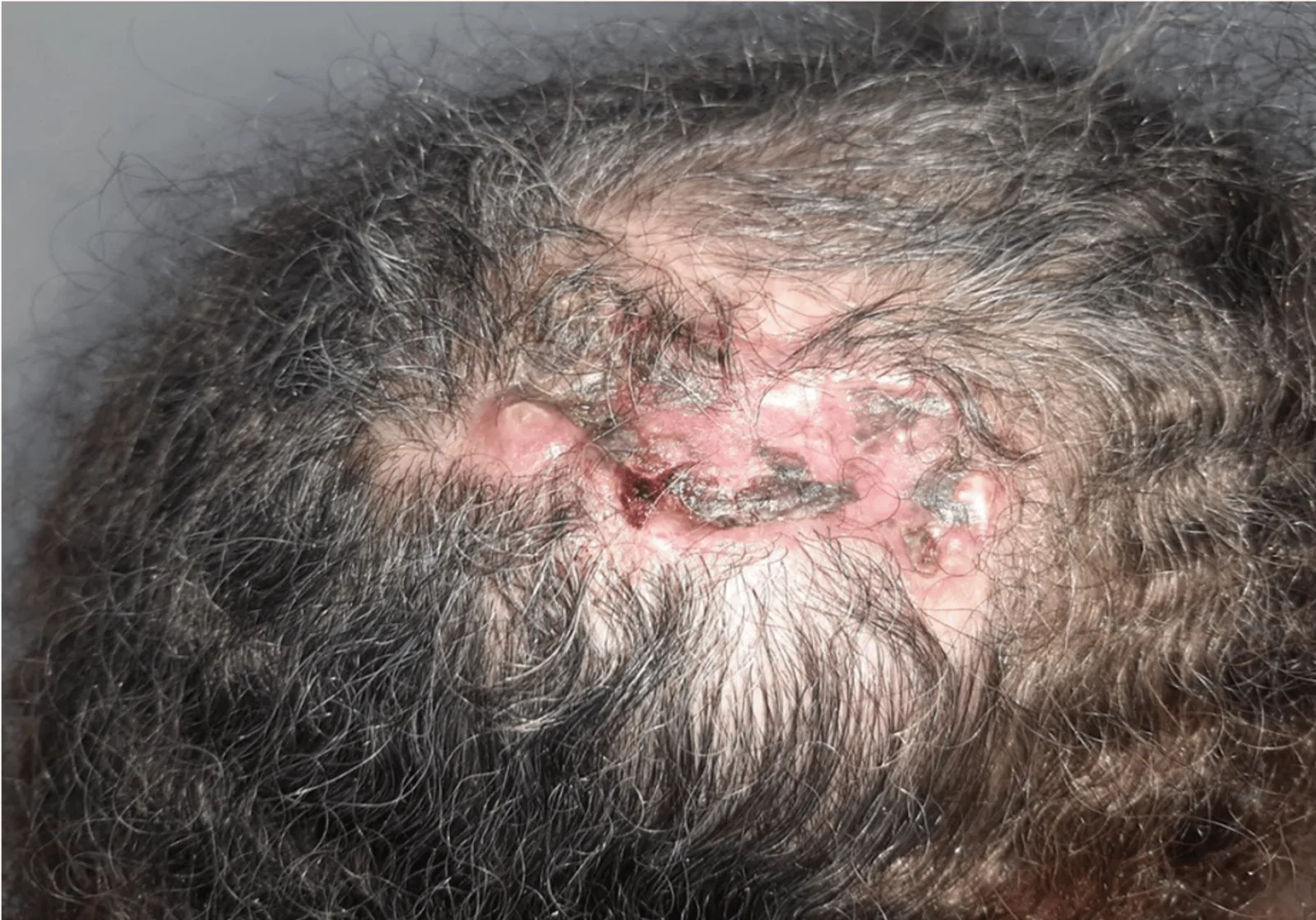
Clinical presentation of the scalp lesion Large ulcerated infiltrated plaque involving the vertex of the scalp

A deep skin biopsy demonstrated a poorly circumscribed dermal neoplasm composed of epithelioid cells arranged in short cords and small nests embedded within a hyalinized stroma. The tumor cells showed moderate nuclear atypia with rare mitotic figures. Immunohistochemical analysis revealed strong expression of CD31 and ERG, whereas CD34 expression was absent (Figure 4).

**Figure 4:**
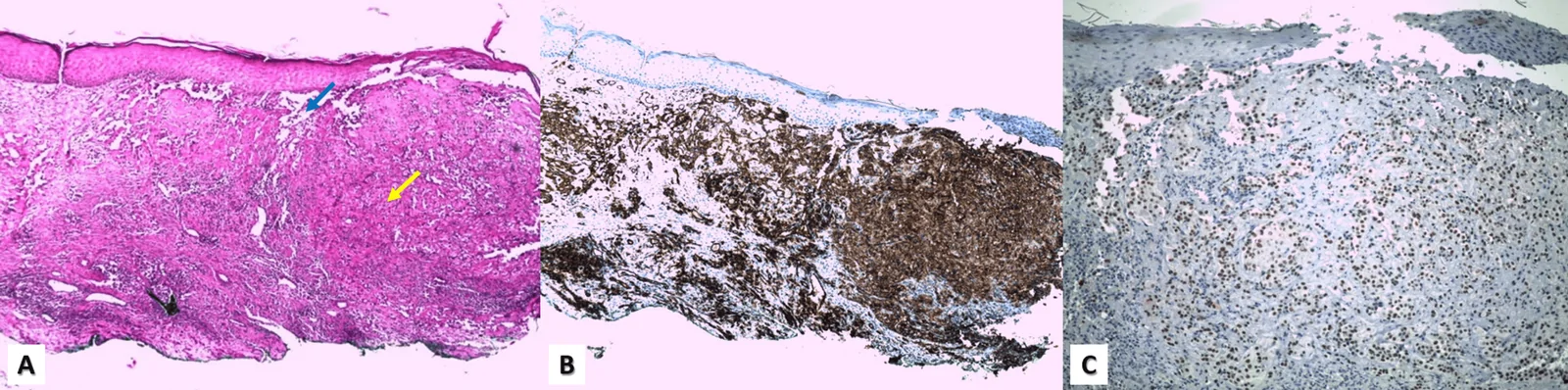
Histopathological and immunohistochemical findings (A) Hematoxylin and eosin staining (×40) showing a dermal proliferation of epithelioid endothelial cells arranged in nests and short cords (yellow arrow) embedded within a characteristic myxohyaline stroma (blue arrow). (B) Immunohistochemical staining showing diffuse membranous positivity for CD31 (×40), confirming endothelial differentiation. (C) Immunohistochemical staining demonstrating diffuse nuclear positivity for ERG (×100), supporting the diagnosis of epithelioid hemangioendothelioma

The Ki-67 proliferation index was approximately 15%. Whole-body 18F-FDG PET/CT confirmed disseminated metastatic disease with pulmonary, hepatic, and osteolytic skeletal metastases (Figure 5).

**Figure 5:**
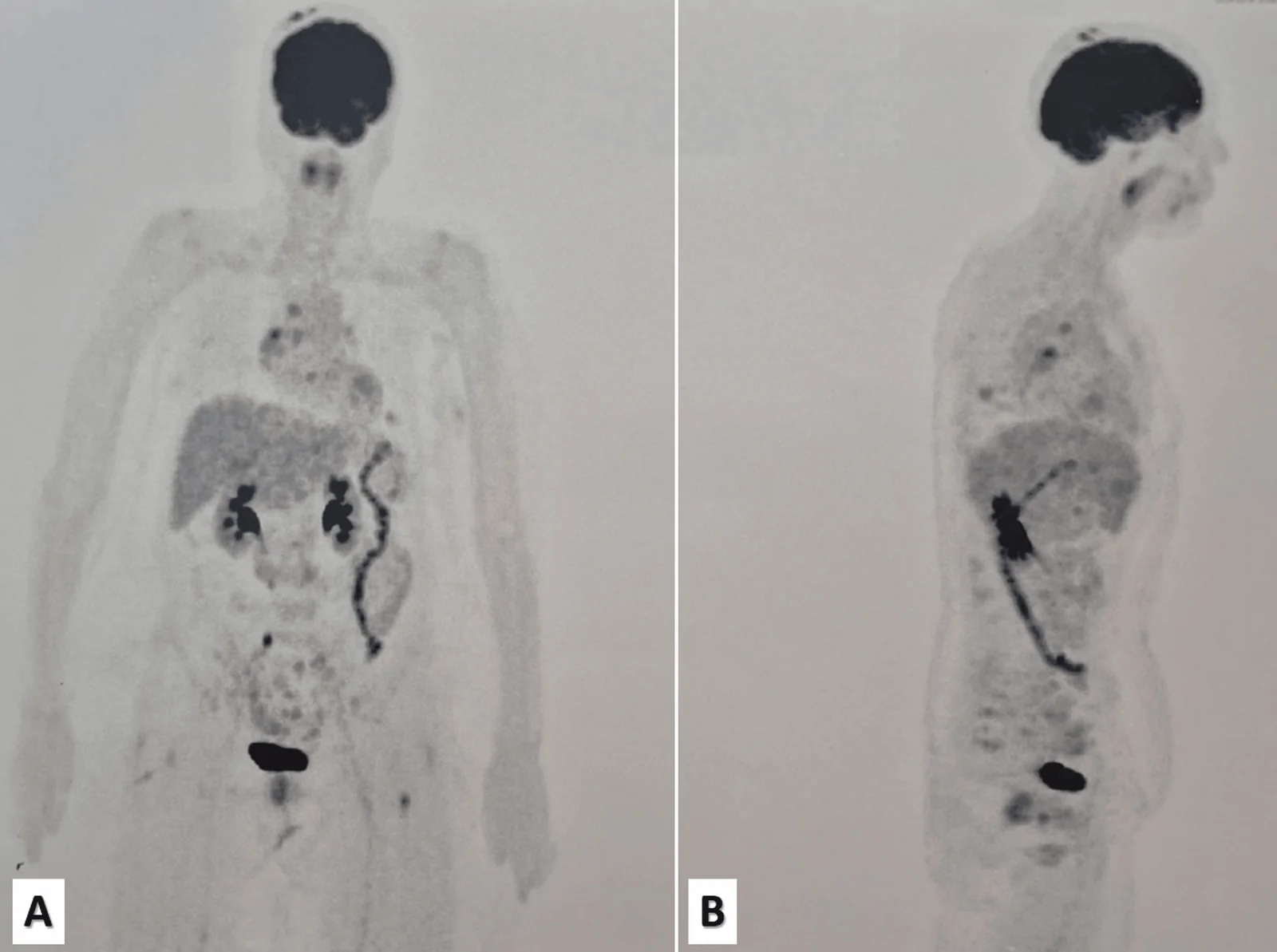
Whole-body 18F-FDG PET/CT MIP (A) Anterior and (B) lateral MIP images demonstrating multifocal FDG-avid lesions involving the scalp, lungs, liver, and skeleton, consistent with disseminated metastatic disease MIP: maximum intensity projection; ^18^F-FDG PET: ^18^F-fluorodeoxyglucose positron emission tomography; CT: computed tomography

Based on the clinicopathological, immunohistochemical, and radiological findings, the diagnosis of primary cutaneous EHE of the scalp with pulmonary, hepatic, and osseous metastases was established. The patient was referred to the Department of Medical Oncology, and systemic chemotherapy with weekly paclitaxel was initiated.

Following six months of weekly paclitaxel therapy, follow-up 18F-FDG PET/CT demonstrated disease progression with the appearance of new pulmonary nodules. Consequently, treatment was switched to doxorubicin. After four months of doxorubicin therapy, the patient remains clinically stable with a preserved general condition. Radiological reassessment has not yet been performed (Figure 6).

**Figure 6:**
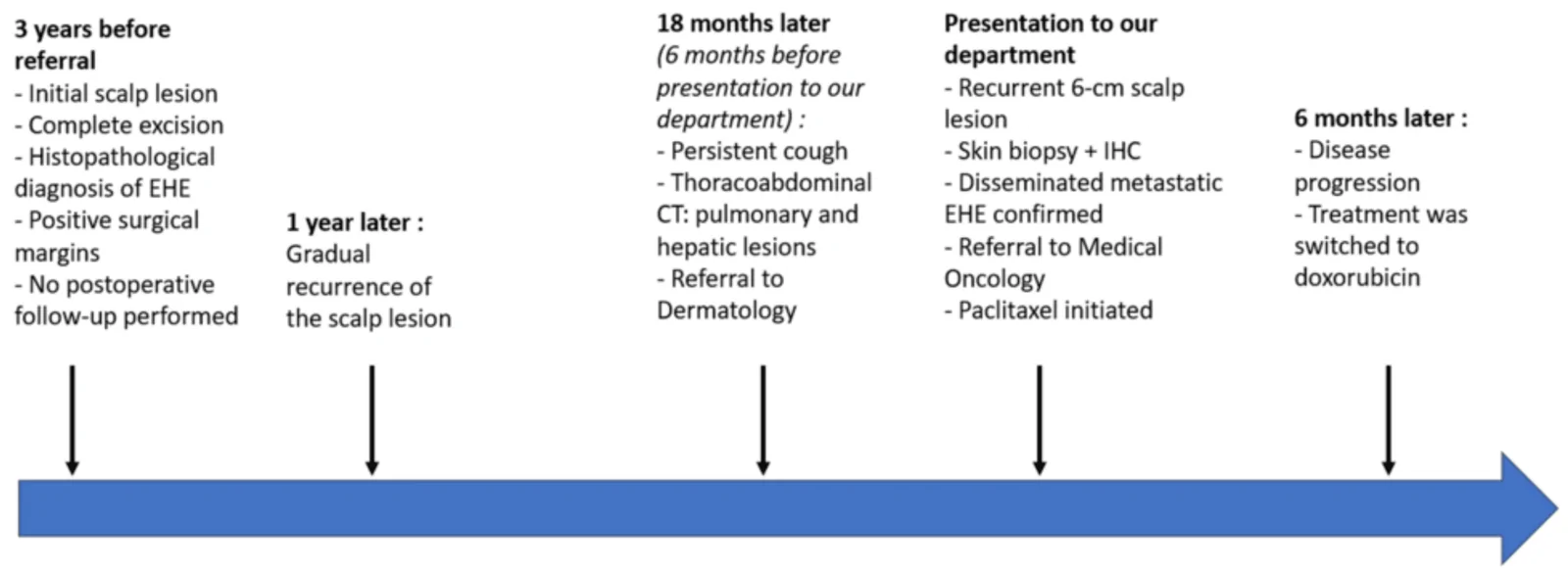
Chronological timeline of the patient's clinical course EHE: epithelioid hemangioendothelioma; CT: computed tomography; IHC: immunohistochemistry

## Discussion

EHE is a rare vascular neoplasm of intermediate malignant potential that occupies a biological spectrum between benign hemangioma and highly aggressive angiosarcoma. It originates from vascular endothelial cells and most frequently involves the liver, lungs, bones, and deep soft tissues, whereas primary cutaneous EHE is exceedingly uncommon [1,2].

The clinical presentation of cutaneous EHE is highly variable and may mimic benign vascular lesions, fibrohistiocytic tumors, or other cutaneous neoplasms, often leading to delayed diagnosis. The extremities are the most frequently affected sites, whereas primary involvement of the scalp is exceptional [2]. Although isolated cases of primary cutaneous scalp EHE associated with lymph node metastasis, metastatic scalp involvement from EHE arising at other sites, and primary calvarial EHE presenting as scalp swelling have been reported, to our knowledge, our case represents the first reported primary cutaneous EHE of the scalp associated with synchronous pulmonary, hepatic, and osseous metastases [3-5].

In the present case, the diagnosis of a primary cutaneous scalp EHE is strongly supported by the initial histopathological diagnosis established from the scalp lesion three years before the detection of visceral metastases. This chronological sequence argues against secondary cutaneous involvement from an occult deep primary tumor. Histologically, the lesion demonstrated epithelioid cells arranged in short cords and small nests embedded within a hyalinized stroma. Immunohistochemistry showed diffuse expression of CD31 and ERG with loss of CD34 expression, a profile consistent with EHE and helpful in excluding several histological mimickers [1,2]. Histologically, the differential diagnosis included epithelioid angiosarcoma, epithelioid hemangioma, epithelioid sarcoma, metastatic carcinoma, and melanoma because these entities may also present as epithelioid neoplasms involving the skin. The characteristic arrangement of epithelioid cells in short cords and small nests embedded within a myxohyaline stroma, together with diffuse CD31 and ERG positivity and loss of CD34 expression, strongly favored the diagnosis of EHE over its principal histological mimickers.

The Ki-67 proliferation index was approximately 15%. Although no universally accepted prognostic cutoff has been established for EHE, higher Ki-67 labeling has been associated with increased proliferative activity and may correlate with a more aggressive clinical course in some studies [3].

Approximately 90% of EHE cases harbor a WWTR1::CAMTA1 gene fusion, whereas a minority of cases harbor a YAP1::TFE3 fusion [6]. Detection of these molecular alterations by fluorescence in situ hybridization or next-generation sequencing may be particularly helpful in diagnostically challenging cases. Molecular testing was not available at our institution and therefore could not be performed. Although this represents a limitation of our report, the diagnosis was supported by the characteristic histopathological and immunohistochemical features.

Complete surgical excision with histologically negative margins remains the treatment of choice for localized disease [1,6,7]. When complete resection cannot be achieved, adjuvant radiotherapy may be considered to improve local disease control [6]. In our patient, the absence of complete excision and subsequent follow-up may have contributed to local persistence of the lesion. Nevertheless, the metastatic evolution probably reflects the intrinsic biological aggressiveness and unpredictable behavior of EHE.

The management of metastatic EHE remains challenging because of the rarity and biological heterogeneity of the disease. No standard systemic treatment has been established. Anthracycline-based chemotherapy, paclitaxel, sirolimus, pazopanib, and other antiangiogenic agents have shown variable and generally modest clinical activity in selected patients [1]. In our patient, weekly paclitaxel was selected as the initial systemic treatment because of its reported activity in vascular sarcomas, its favorable toxicity profile, and its immediate availability at our institution. More recently, encouraging results have been reported with taxane-based chemotherapy in patients with advanced EHE, further supporting this therapeutic approach [1,8]. However, after six months of treatment, disease progression was documented by follow-up 18F-FDG PET/CT, prompting a switch to doxorubicin. At the latest follow-up, the patient remained clinically stable after four months of doxorubicin therapy, with radiological reassessment still pending. Given the rarity and unpredictable clinical behavior of metastatic EHE, treatment should be individualized within a multidisciplinary setting.

Although EHE generally exhibits a less aggressive clinical behavior than angiosarcoma, it retains a considerable risk of both local recurrence and metastatic spread. In head and neck lesions, recurrence and metastasis have been reported in up to 44% and 38% of cases, respectively [3]. These findings underscore the importance of prolonged clinical surveillance, even after apparently adequate treatment.

## Conclusions

Primary cutaneous EHE of the scalp is an exceptionally rare vascular neoplasm with an unpredictable clinical course and the potential for late distant metastatic dissemination. This case highlights the importance of early recognition, careful clinicopathological correlation supported by immunohistochemistry, and complete surgical excision with histologically negative margins whenever feasible. Given its metastatic potential and risk of recurrence, long-term surveillance and multidisciplinary management are essential to optimize patient outcomes.
